# Level of advanced oxidation protein products is associated with subclinical atherosclerosis

**DOI:** 10.1186/s12872-021-02451-2

**Published:** 2022-01-08

**Authors:** Zsolt Bagyura, Angéla Takács, Loretta Kiss, Edit Dósa, Réka Vadas, Tin Dat Nguyen, Elek Dinya, Pál Soós, Zsolt Szelid, Orsolya Láng, Éva Pállinger, László Kőhidai, Béla Merkely

**Affiliations:** 1 Heart and Vascular Center, Semmelweis University, Városmajor utca 68, Budapest, 1122 Hungary; 2 Department of Genetics, Cell- and Immunobiology, Semmelweis University, Nagyvárad tér 4, Budapest, 1089 Hungary; 3 Institute of Digital Health Sciences, Semmelweis University, Ferenc tér 15, Budapest, 1094 Hungary

**Keywords:** Carotid intima-media thickness, Advanced oxidation protein products, Oxidative stress

## Abstract

**Background:**

Oxidative stress is an important factor in the pathomechanism of atherosclerosis. Advanced oxidation protein products (AOPPs) are considered markers of oxidative stress. Thickening of the carotid intima-media layers indicates subclinical atherosclerosis and can be detected by carotid ultrasound.

**Objective:**

Our aim was to examine the association between carotid intima-media thickness (CIMT) and the level of AOPPs.

**Methods:**

Carotid duplex scans and measurements of AOPPs were performed on 476 participants of a cardiovascular population study. The presence of conventional cardiovascular risk factors was investigated with a questionnaire, physical examination, and laboratory tests.

**Results:**

There was a positive correlation between maximum CIMT and the level of AOPPs only in the male population (r = 0.219, *p* = 0.033). Multivariate analysis has revealed that the association between AOPPs and mean or maximum CIMT was independent of cardiovascular risk factors (OR = 1.458, *p* = 0.004, and OR = 2.038, *p* < 0.001).

**Conclusions:**

Among males, the elevated level of AOPPs as a marker of oxidative stress may signal the existence of early atherosclerotic alterations.

## Background

Cardiovascular diseases (CVDs) are the main independent causes of morbidity and mortality worldwide, accounting for over 4 million deaths per year in the European Union [1]. In most cases, CVDs are due to atherosclerosis of the medium- and large-sized arteries [2]. Therefore, early detection of atherosclerosis in a subclinical stage has great importance in personalized risk assessment. The abnormal thickening of the carotid intima-media, which can be precisely measured by carotid ultrasonography, is a commonly accepted marker of subclinical atherosclerosis [3]. Thickening of the carotid intima-media layers is associated with total plaque burden according to histological studies [4]. Epidemiologic studies have shown a positive correlation between classical cardiovascular risk factors [e.g. hypertension [5], diabetes mellitus [6], and hypercholesterolemia [7]] and the abnormal changes of the carotid intima-media thickness (CIMT). A similar association exists between non-classical cardiovascular risk factors (e.g. elevated serum uric acid levels) and the abnormal CIMT [8]. Even the systematic use of CIMT measurement for risk assessment is no longer recommended in daily clinical routine by the current clinical guidelines [9] due to the technical difficulties, correctly performed carotid intima-media thickness measurement is also a known predictor of cardiovascular events and coronary heart disease (CHD) [10].

Atherosclerosis is considered a multifactorial chronic inflammatory disease, and its pathogenesis involves oxidative stress [11, 12]. In vivo, advanced oxidation protein products (AOPPs) are the end-products of a reaction between plasma albumin and chlorinated oxidants, such as hypochlorous acid, and formed through the myeloperoxidase [13]. Nonfunctional proteins contain di-tyrosine cross-links [14]. Once AOPPs are formed, they can lead to tissue damage as a result of an imbalance between the antioxidant and pro-oxidant levels and to the accumulation of reactive oxygen species (ROS), which are strong oxidizing agents.

Detection of AOPPs is usually a consequence of the aforementioned impaired balance of the redox homeostasis. As the measurement of ROS is difficult due to their diverse form and short half-life, AOPPs as biomarkers of oxidation-mediated protein damage can be used as reliable indicators reflecting oxidative stress status [15]. The association between AOPPs as oxidative stress markers and atherosclerosis has been already investigated, and it was shown that atherogenic risk is elevated in AOPPs-treated mice [16]. However, whether the association exists independently of conventional risk factors in an asymptomatic population is not known. Our aim was to examine oxidative stress status according to the level of AOPPs and its association with CIMT as a marker of subclinical atherosclerosis in a cross-sectional study.

## Materials and methods

### Participant selection

Budakalász Health Examination Survey, a cross-sectional voluntary cardiovascular screening program targeting the adult population (> 20 years, around 8000 inhabitants) of a Central-Hungarian town (Budakalász) was performed in 2011–2013 [17]. Medical history with special attention to CVD-related signs and symptoms, lifestyle, and family history were recorded by an experienced physician. Anthropometric parameters (height, weight) rounded to the nearest 0.1 cm and 0.1 kg were measured in the standing position while participants were wearing light indoor clothing without shoes.

Routine laboratory tests were performed in our Institution’s Central Laboratory with rigorous quality control. The concentration of lipid fractions was measured by using a colorimetric assay (Roche Diagnostics Ltd., Mannheim, Germany).

**Fig. 1:**
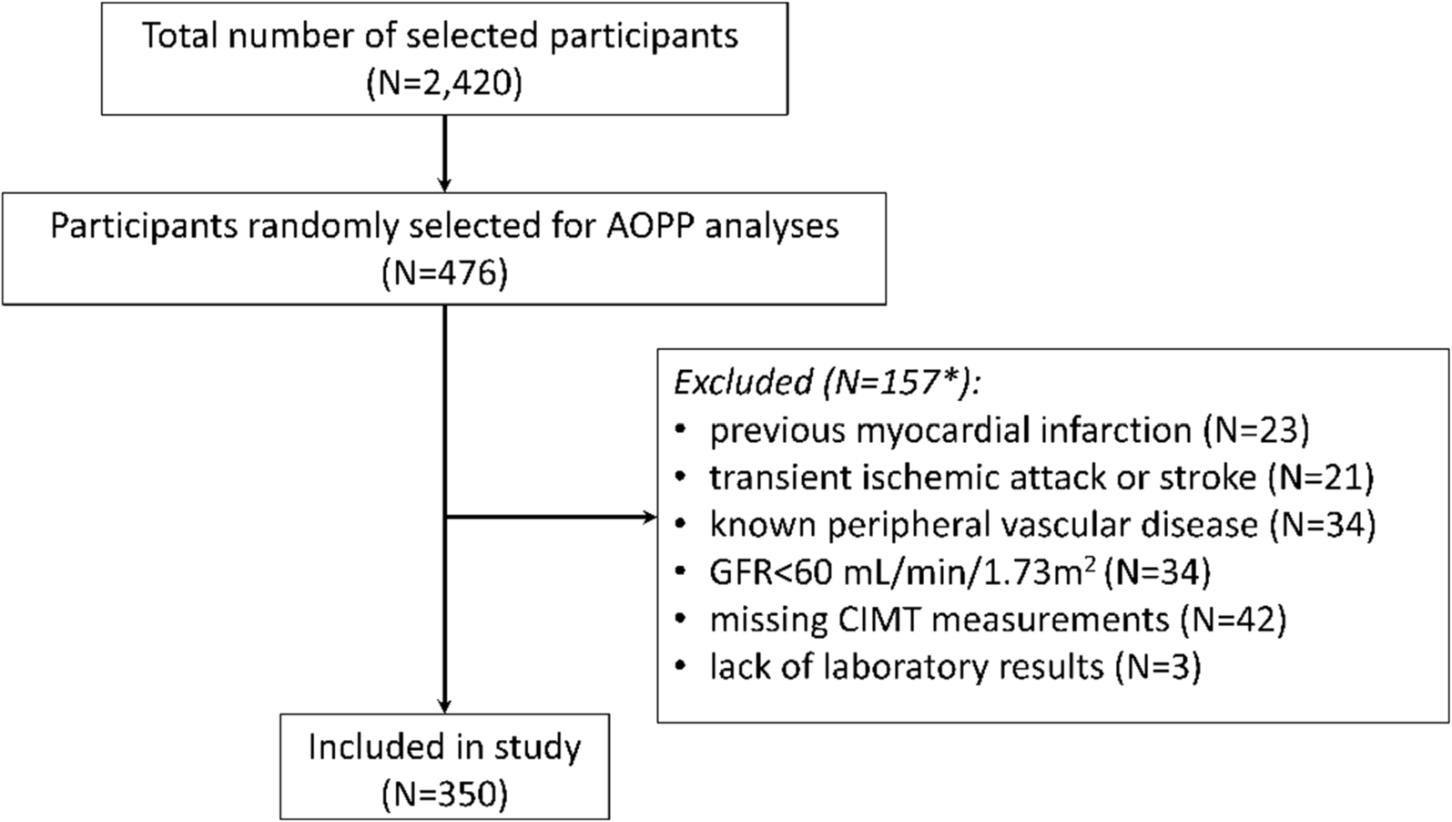
Flow chart of participant selection. *A total of 31 participants fulfilled 2 or more exclusion criteria

The participation rate in the Budakalász Health Examination Survey was around 30% (N = 2420) of the eligible total population. Measurement of AOPPs was performed in 476 participants selected randomly. Participants with the following cardiovascular history were excluded from further analysis: 23 participants (4.8%) with previous myocardial infarction, 21 participants (4.4%) with transient ischemic attack or stroke, 34 participants (7.1%) with known peripheral vascular disease, and 34 participants (7.1%) with glomerular filtration rate (GFR) < 60 mL/min/1.73 m^2^. Three participants (0.6%) due to the lack of laboratory results and 42 participants (8.7%) due to the missing CIMT measurements were also excluded (Fig. 1). As a participant could have more than one exclusion criterion, overall 350 participants were included in the study.

### Advanced oxidation protein products measurement

Blood samples were drawn in ethylenediaminetetraacetic acid (EDTA) treated tubes and then subjected to centrifugation. Plasma samples were stored at − 80 °C until the determination of AOPPs. Analysis of the unknown levels of AOPPs in the plasma samples was based on a spectrophotometric detection according to Witko-Sarsat et al. [18] and Taylor et al. [19]. The principle of the assay is a colorimetric reaction between plasma proteins and chlorinated oxidants (e.g. chloramine-T). All assays were carried out on triplicates. First, plasma samples were diluted 1:10 in phosphate-buffered saline (PBS), then 300 µL of the diluted plasma samples were added to each well of the microtiter plate (Sarstedt AG & Co. KG, Nümbrecht, Germany). For optimal accuracy, Chloramine-T hydrate (Sigma Ltd. St. Louis, MO, USA) standard solutions (300 µL; 0–100 µM) were also placed in the same plate and PBS served as a sample blank. Fifteen micro litter 1.16 M potassium iodide (KI; Sigma Ltd. St. Louis, MO, USA) was added to all wells, followed by incubation at room temperature for 2 min. After all, 30 µL glacial acetic acid (Sigma Ltd. St. Louis, MO, USA) was also added to all wells. Prior to reading the absorbance at 340 nm, plates were centrifuged at 5800 g for 5 min, and 230 µL precipitated-protein-free supernatant was transferred to a new 96-well microplate (Sarstedt AG & Co. KG, Nümbrecht, Germany). In all cases, the absorbance of the sample blank was subtracted from the sample wells before further analysis. The calculated chloramine-T equivalent concentrations of AOPPs were normalized to the total protein of each patient and expressed in µM/g protein.

### Diagnostic criteria and clinical definitions

Medical history was regarded as positive for hypertension and hyperlipidemia if documented. The following cut-off points were used: > 140 mmHg systolic and/or > 90 mmHg diastolic for elevated blood pressure, and 5.2 mmol/L for total cholesterol, 3.4 mmol/L for low-density lipoprotein cholesterol (LDL-C), 1.7 mmol/L for triglyceride, and 1.1 mmol/L or below (females) and 0.9 mmol/L or below (males) for high-density lipoprotein cholesterol (HDL-C). Current and former regular smokers, who quit smoking at least one year before and smoke regularly at least for one year were both considered smokers. Body mass index (BMI) was calculated using the Quetelet's form.

### Carotid intima-media thickness measurements

High-quality, B-mode longitudinal ultrasonography images (VIVID I, GE Healthcare, Wauwatosa, USA) of the common carotid artery (CCA) were acquired at end-diastole on both sides. Following the concepts of the 2012 Mannheim Consensus [20], a 10 mm length of a straight arterial segment without plaques was marked out on the far wall of the CCA at least 5 mm below the bifurcation for intima-media thickness measurement. All markings were performed by the same person. Then, a semiautomatic software (GE EchoPAC BT11, GE Healthcare, Wauwatosa, USA) calculated and recorded the maximum and mean values of CIMT. The maximum value was used for analysis, and the values of the left and right sides were averaged.

### Statistical analysis

SPSS for Windows, version 25.0 (IBM, Armonk, NY, USA) was used for statistical analyses. A post hoc power analysis was conducted using an online calculator developed by MGH Biostatistics Center. The sample size of 350 was used for the statistical power analyses. The significance level used for this analysis was *p* < 0.05. The standard deviation of the dependent variable (maximal CIMT) was 0.16, the standard deviation of the independent variable (AOPP) was 0.08, the minimal detectable difference entered was 0.36. The post hoc analyses revealed that the probability is 91% that the study detects a relationship between the independent and the dependent variables. Distribution of AOPP in the population was tested by the Kolmogorov–Smirnov test and showed normal distribution (*p* = 0.065). Continuous variables were expressed as means with standard deviation (SD) or medians with interquartile ranges (IQRs) as appropriate, depending on the distribution of the values, whereas categorical variables were expressed as numbers (percentages). Comparisons of means, medians, and proportions were performed with Student’s *t* test, Mann–Whitney U-test, and Chi-square test, respectively. Spearman’s linear correlation was used to test the association between AOPPs and CIMT. Generalized linear model (GLZ) analysis was performed to screen the significant predictor risk factors adjusted for age, and gender. All analyses were two-tailed, and *p* < 0.05 was considered significant.

## Results

### Patient characteristics

Overall, 350 participants were included in the final analysis. The baseline clinical characteristics of the 350 participants are shown in Table 1.

**Table 1:** Baseline clinical characteristics

Characteristics	All participants	Female (N = 205)	Male (N = 145)	*p* value
Age (years), mean (SD)	59.33 (10.31)	60.86 (9.86)	57.51 (10.85)	0.016*
LDL-C (mmol/L), mean (SD)	3.51 (0.99)	3.47 (0.96)	3.55 (1.05)	0.57
HDL-C (mmol/L), mean (SD)	1.53 (0.48)	1.65 (0.47)	1.33 (0.39)	< 0.001*
Triglycerides (mmol/L), mean (SD)	2.41 (1.41)	2.28 (1.45)	2.66 (1.58)	0.064
Cholesterol (mmol/L), mean (SD)	5.81 (1.1)	5.84 (1.05)	5.75 (1.16)	0.54
HbA1c (%), mean (SD)	5.76 (0.53)	5.79 (0.49)	5.72 (0.57)	0.367
BMI (kg/m^2^), mean (SD)	28.78 (5.42)	28.84 (6.03)	28.54 (4.34)	0.681
Hypertension, N (%)	122 (33.9)	67 (50.8)	55 (57.9)	0.345
DM, N (%)	22 (6.3)	10 (7.6)	12 (12.6)	0.265
Smoking, N (%)	21 (6)	10 (7.6)	11 (11.6)	0.356
Dyslipidemia, N (%)	100 (28.5)	62 (47)	38 (40)	0.343
Creatinine (µmol/L), mean (SD)	75.98 (18.33)	75.43 (15.93)	76.81 (18.87)	0.555
Serum uric acid (mg/dL), mean (SD)	324.53 (77.69)	303.89 (67.55)	357.55 (82.69)	< 0.001*
Beta-blockers, N (%)	52 (14.9)	34 (25.8)	18 (18.9)	0.264
ASA, N (%)	23 (6.6)	11 (8.3)	12 (12.6)	0.373
ARB, N (%)	35 (10)	14 (10.6)	21 (22.1)	0.025*
ACEI, N (%)	98 (28)	59 (28.8)	39 (36.9)	0.719
Diuretics^#^, N (%)	15 (4.3)	7 (5.3)	8 (8.4)	0.421
Lipid-lowering medications^#^, N (%)	46 (13.1)	26 (19.7)	20 (21.1)	0.868
Antidiabetics^#^, N (%)	27 (7.7)	17 (10.2)	15 (13.6)	0.434
Normalized AOPPs (µM/g) mean (SD)	0.36 (0.08)	0.36 (0.07)	0.38 (0.07)	0.044*
Normalized AOPPs (µM/g) median (IQR)	0.36 (0.12)	0.35 (0.12)	0.38 (0.12)	0.33
Maximal CIMT (mm), median (IQR)	0.8 (0.22)	0.8 (0.22)	0.8 (0.24)	0.839
Maximal CIMT (mm), mean (SD)	0.83 (0.168)	0.83 (0.16)	0.83 (0.17)	0.857
Mean CIMT (mm), mean (SD)	0.68 (0.12)	0.68 (0.12)	0.68 (0.13)	0.713
Mean CIMT (mm), median (IQR)	0.68 (0.15)	0.68 (0.16)	0.65 (01)	0.616
Carotid plaque, N (%)	78 (22.3%)	45 (22)	33 (22.5)	0.897

Mean ± SD or median (IQR) represented the continuous variables, and numbers (proportions) the categorical variables. Abbreviations: ACEI, angiotensin-converting enzyme inhibitor; AOPP, advanced oxidation protein products; ARB, angiotensin receptor blockers; ASA, acetylsalicylic acid; BMI, body mass index; CIMT, carotid intima-media thickness; DM, diabetes mellitus; HbA1c, hemoglobin A1c; HDL-C, high-density lipoprotein cholesterol; IQR, interquartile range; LDL-C, low-density lipoprotein cholesterol; SD, standard deviation; #oral antidiabetics, *significant values

The mean age of the participants was 59.33–0.31 years. Fifty-seven point 5% were males, all patients belonged to the Caucasian race. The characteristics of female and male patients were compared (Table 1). We found, that there was no significant difference in terms of the general cardiovascular risk factors, except for HDL-C and serum uric acid. Males had lower HDL-C (1.65 vs. 1.33, *p* < 0.001) and higher serum uric acid levels (303.89 vs. 357.55, *p* < 0.001), although the females were significantly older than the males (60.86 vs. 57.51, *p* = 0.016). History of dyslipidemia and diabetes mellitus, as well as serum LDL-C, triglyceride, cholesterol levels did not significantly differ between the two groups. A list of chronic medications is also shown in Table 1. Of all patients studied, the three most common medications were angiotensin-converting enzyme inhibitor (ACEI) (28%), beta-blockers (14.9%) and lipid-lowering medications (13.1%). There was a significant difference found between the groups regarding the angiotensin receptor blockers (ARB) (10.6% vs. 22.1%, *p* = 0.025) therapy they were receiving.

The mean level of the normalized AOPPs was 0.36 µMg ± 0.07 in females, and it was significantly increased in male participants (0.38 µMg ± 0.07; *p* = 0.044). Further analysis showed that there was no statistically significant difference in the CIMT values between the two groups.

#### Correlation of AOPP with risk factors

In the whole population, we observed a significant negative correlation between the value of the normalized mean AOPP with HDL-C (r =  − 0.232, *p* < 0.001) (Table 2) and positive correlation between normalized mean AOPP and LDL-C (r = 0.162, *p* < 0.016). While this trend existed in females (HDL-C: r =  − 0.235, *p* = 0.007; LDL-C (r = 0.162, *p* = 0.038), in males only correlation of HDL-C and mean AOPP was noticed (HDL-C: r =  − 0.202, *p* = 0.049). Moreover, BMI correlated positively with normalized mean AOPP (r =  − 0.209, *p* = 0.042) only in males. Maximum CIMT and mean plasma level of AOPPs showed a significant positive correlation (r = 0.184, *p* = 0.006) in the total study population (Table 2). This tendency could also be recognized in males (r = 0.219, *p* = 0.033), but not in females. In contrast, there was no significant correlation between AOPP and mean CIMT in the univariate analysis.

**Table 2:** Correlation of normalized mean AOPP level with risk factors

Parameter	All participants		Female		Male	
	Correlation coefficient (r)	*p* value	Correlation coefficient (r)	*p* value	Correlation coefficient (r)	*p* value
Age	0.025	0.705	0.081	0.354	− 0.003	0.974
BMI	− 0.105	0.114	− 0.043	0.628	− 0.209	0.042*
HDL-C	− 0.232	< 0.001*	− 0.235	0.007*	− 0.202	0.049*
LDL-C	0.162	0.016*	0.183	0.038*	0.107	0.313
HbA1c	− 0.083	0.217	− 0.069	0.438	− 0.057	0.587
Serum creatinine	0.035	0.596	0.012	0.890	0.067	0.587
Serum uric acid	0.078	0.242	0.104	0.237	− 0.05	0.623
Maximum CIMT	0.184	0.006*	0.166	0.058	0.219	0.033*
Mean CIMT	0.086	0.195	0.084	0.340	0.101	0.328

AOPPs, Advanced oxidation protein products; BMI, body mass index; CIMT carotid intima-media thickness; HbA1c, hemoglobin A1c; HDL-C, high-density lipoprotein cholesterol; LDL-C, low-density lipoprotein cholesterol; *p* and r values are for Spearman’s correlation; *significant values

### Multivariate analysis

Generalized linear model analysis of mean and maximum CIMT was performed with adjustment for age and AOPPs. In this model AOPPs were significantly associated with both mean and maximal CIMT in males (OR = 1.92, *p* = 0.02 and OR = 1.68, *p* = 0.001; respectively) but not in females.

Further adjustments have been performed for known cardiovascular risk factors such as smoking status, hypertension, dyslipidemia, diabetes mellitus, HDL-C level, LDL-C level, HbA1c level and for ARB therapy (Tables 3 and 4). Multivariate analysis revealed that age was significantly associated with mean and maximum CIMT in the total study population, regardless the gender. The level of AOPP was significantly associated with mean and maximum CIMT in males (OR = 1.458, *p* = 0.004; OR = 2.038, *p* < 0.001, respectively) only. (Tables 3 and 4).

**Table 3:** Multivariate analysis of risk factors related to mean CIMT

Mean CIMT	Females				Males			
Parameter	OR	*p* value	95% CI		OR	*p* value	95% CI	
			Lower	Upper			Lower	Upper
Age	1.006	< 0.001*	1.182	2.144	1.008	< 0.001*	1.006	1.011
AOPPs	0.980	0.879	0.753	1.274	1.458	0.004*	1.125	1.889
Smoker	1.041	0.136	0.987	1.098	1.036	0.191	0.982	1.093
Hypertension	1.029	0.168	0.988	1.071	0.976	0.370	0.925	1.03
Dyslipidemia	0.998	0.907	0.960	1.036	0.936	0.001*	0.901	0.972
Diabetes	1.014	0.656	0.952	1.081	1.035	0.293	0.971	1.104
BMI	0.999	0.371	0.996	1.002	1.005	0.079	0.999	1.01
HDL-C	0.973	0.183	0.934	1.013	1.007	0.752	0.964	1.052
LDL-C	0.944	0.492	0.975	1.012	1.018	0.049*	1.001	1.035
HbA1c	0.944	0.257	0.953	1.036	1.032	0.079	0.996	1.069
ARB therapy	0.956	0.257	0.885	1.033	0.967	0.164	0.922	1.015

AOPPs, Advanced oxidation protein products; ARB, angiotensin receptor blockers; BMI, body mass index; HbA1c, hemoglobin A1c; HDL-C, high-density lipoprotein cholesterol; *significant values

**Table 4:** Multivariate analysis of risk factors related to maximum CIMT

Maximum CIMT	Females				Males			
Parameter	OR	*p* value	95% CI		OR	*p* value	95% CI	
			Lower	Upper			Lower	Upper
Age	1.008	< 0.001*	1.32	2.68	1.009	< 0.001*	1.006	1.013
AOPPs	1.184	0.347	0.833	1.683	2.038	< 0.001*	1.441	2.883
Smoker	1.034	0.3	0.97	1.102	1.013	0.714	0.944	1.088
Hypertension	1.019	0.505	0.964	1.078	0.988	0.759	0.918	1.088
Dyslipidemia	0.972	0.288	0.921	1.025	0.927	0.004*	0.88	0.976
Diabetes	1.043	0.307	0.962	1.13	1.081	0.036*	1.005	1.162
BMI	1	0.935	0.995	1.004	1.008	0.021*	1.001	1.015
HDL-C	0.957	0.124	0.906	1.012	1.002	0.413	0.970	1.078
LDL-C	0.987	0.268	0.963	1.011	1.034	0.003*	1.011	1.057
HbA1c	0.958	0.089	0.911	1.007	1.038	0.134	0.989	1.089
ARB therapy	0.949	0.322	0.855	1.053	0.953	0.166	0.890	1.02

We have performed a multivariate analysis with carotid plaque as a dependent variable adjusted for age and AOPPs for both genders. In these models, AOPPs were not significantly associated with carotid atherosclerosis

## Discussion

In this study, we presented an independent association of AOPPs with mean or maximum CIMT in males in an asymptomatic Caucasian population. In logistic regression analyses, AOPPs were independent predictors and showed significantly increased odds (OR = 1.458 and OR = 2.038) for the presence of higher mean and maximal CIMT values in males but not in females.

Ultrasound measurement of carotid intima-media thickness is a valuable tool to assess the vascular status, as the abnormal CIMT value is a known biomarker of subclinical atherosclerosis. Although in the literature there is no general definition of abnormal CIMT, it has been indicated that mean CIMT above 0.9 mm correlates with atherosclerosis [21], traditional cardiovascular risk factors [22–24] as well as increased prevalence and incidence of CVD [22]. The analysis of mean and maximum CIMT in our study did not confirm any significant gender differences in carotid intima-media thickness between females and males.

The level of AOPPs measured in our study population was similar to the results of other population-based studies, including clinically healthy participants [25]. We found that there was a significantly higher level of normalized AOPPs in male participants compared to females (0.38 vs. 0.36, *p* = 0.044) this result is in line with other studies [26]. The level of AOPP significantly correlated with maximum CIMT (r = 0.184, *p* = 0.006) in the whole population. Analyzing this correlation by gender revealed that AOPP and maximal CIMT correlated in the male population (r = 0.219 *p* = 0.033), but this could not be observed in females. In contrast, no significant correlation was detected related to mean CIMT in the univariate analysis.

The association between CIMT and other markers of oxidative stress was investigated in various studies. Ashfaq et al. revealed that glutathione redox state, an in vivo measure of intracellular oxidative stress, correlated with mean CIMT in a healthy population [27]. Yagi et al. found that biological antioxidant potential negatively correlated with CIMT, which suggests a positive association of oxidative stress with CIMT [28]. In a pilot study, Seyedsadjadi et al. showed that a comprehensive redox balance lifestyle score was significantly associated with the risk for increased CIMT [29].

After multivariate adjustment for cardiovascular risk factors, age was a weak, but independent predictor for both mean and maximum CIMT in females and males. In the male population, both mean and maximum CIMT correlated with AOPPs (OR = 1.458, *p* = 0.004 and OR = 2.038, *p* < 0.001, respectively) as further variables, such as LDL-C level and dyslipidemia but not in females.

It is generally accepted that there are gender differences in cardiovascular diseases. Female gender is associated with lower prevalence of CVD [30] and cardiovascular diseases tend to develop later in women than in men [31] and this is known to be associated with the protective antioxidant effects of female hormones [32]. AOPP levels seem to be associated with gender according to our and other’s results [26].

The level of AOPPs reflects the oxidative stress state in the extracellular space. Oxidative stress may lead to atherosclerosis through several mechanisms. Reactive oxygen species overproduction can cause impaired nitric oxide synthesis, and the function is associated with endothelial dysfunction that is an important factor in the development of atherosclerosis [33]. Also, AOPPs are transported to the subendothelial space where they enhance oxidative stress and inflammation, thus triggering plaque formation [34]. Surprisingly, in our study no correlation was found between AOPP and carotid plaque formation. An increase in the level of AOPPs has already been reported in several diseases such as uremia [35], diabetes mellitus [36], rheumatoid arthritis [37], and coronary artery disease [38]. In a study by Piwowar et al., the level of AOPPs was elevated in diabetic patients and associated with macroangiopathy in this patient group [39]. Liu et al. reported a causal relationship between chronic accumulation of AOPPs and atherosclerosis in an animal model [40].

According to the results of Sanchez et al., the level of AOPPs is associated with cardiovascular risk factors such as BMI and lipid profiles in apparently healthy young adults [41]. Previous studies of patients with renal disease demonstrated that AOPPs were reliable markers of the oxidant-mediated protein damage in hypertriglyceridemia, CHD, and atherosclerosis. Kocak et al. investigated the association between CIMT and AOPPs in peritoneal dialyzed (PD) patients and found that the level of AOPPs was higher in PD patients when compared to controls [42]. Also, AOPPs level correlated with CIMT in the PD group. In contrast to our results, they found no association of AOPP and CIMT in non-dialyzed controls. Of note, the population they analyzed was approximately 20 years younger on average than ours and none of the patients were hypertensive, hyperlipidemic or active smokers unlike in our general study population without clinical signs of atherosclerosis but with the presence of risk factors. Despite all this, we have not found other studies that investigated AOPPs in an asymptomatic population of such a sample size as ours.

To the best of our knowledge, this is the first study demonstrating an independent positive association between the plasma level of AOPPs and the value of CIMT in an asymptomatic population and its’s gender difference.

Our study has some limitations. As the participation in the study was on a voluntary basis, there is the possibility that subjects with known hypertension or other factors associated with subclinical atherosclerosis and elevated CIMT had a greater propensity to participate. Secondly, due to the cross-sectional study type, a cause-and-effect relationship cannot be identified. Therefore, we cannot conclude that CIMT increased because of oxidative stress, the namely elevated level of AOPPs.

In conclusion, in a male population without previous cardiovascular events, mean and maximum CIMT were associated with the elevated level of AOPPs.

## Conclusion

In this study, we found that the elevated level of AOPPs in the male population may be used as an early marker of atherosclerotic alterations.

## Data Availability

The datasets generated during and/or analyzed during the current study are available from the corresponding author on reasonable request.

## Abbreviations

AOPP: Advanced oxidation protein products
ARB: Angiotensin receptor blocker
ASA: Acetylsalicylic acid
BMI: Body mass index
CCA: Common carotid artery
CHD: Coronary heart disease
CIMT: Carotid intima-media thickness
CVD: Cardiovascular diseases
DM: Diabetes mellitus
EDTA: Ethylenediaminetetraacetic acid
HbA1c: Hemoglobin A1c
HDL-C: High-density lipoprotein cholesterol
GLZ: Generalized linear model
GFR: Glomerular filtration rate
LDL-C: Low-density lipoprotein cholesterol
PD: Peritoneal dialyzed
ROS: Reactive oxygen species
